# Role of inflammatory and hematological markers in predicting the complicated and perforated acute appendicitis in pediatric population: A cohort study

**DOI:** 10.1016/j.amsu.2022.103524

**Published:** 2022-04-01

**Authors:** Syed Jawad Haider Kazmi, Shaesta Tabassum, Muhammad Sohaib Asghar, Muhammad Ali Siddiqui, Avinash, Farah Yasmin, Muhammad Junaid Tahir, Yumnah Aziz, Tooba Ahmed Kirmani, Mansoor Ahmed

**Affiliations:** a Department of Emergency Medicine, Liaquat National Hospital and Medical College, Karachi, Pakistan; b Department of Internal Medicine, Dow University of Health Sciences, Karachi, Pakistan; c Department of Internal Medicine, Liaquat National Hospital and Medical College, Karachi, Pakistan; d Department of General Surgery, Liaquat National Hospital and Medical College, Karachi, Pakistan; e Department of Internal Medicine, Lahore General Hospital, Lahore, Pakistan; f Department of Internal Medicine, Chandka Medical College, Larkana, Pakistan

**Keywords:** Appendix, Appendicitis, Diagnosis, Complications, Investigations

## Abstract

**Introduction:**

It is important to promptly assess the severity of appendicitis since late diagnosis can proceed towards perforation, peritonitis and sepsis. The main objective of this study is to decipher the ability of CRP, TLC and neutrophils in acute appendicitis to predict complications and perforation in pediatric age group.

**Methods:**

This cohort study was conducted in the Pediatric Surgery Department of Liaquat National Hospital, Karachi. It included all the patients diagnosed and operated on for acute appendicitis within the pediatric age group.

**Results:**

The median (IQR) age of study population was 9 (7–11) years, the majority of which fell into 6–12 years of age group with 70% males. Histopathology came out positive in 127 individuals. Out of those 127 patients, 45.9% (n = 62) had simple appendicitis and 48.1% (n = 65) had complicated appendicitis (n = 65), while 37 (27.4%) had shown perforation. The majority of individuals had suppurative appendicitis on histopathology (35.6%). On receiver operating characteristic (ROC) analysis, CRP has the highest specificity for complicated appendicitis and the highest positive likelihood ratio for both complicated and perforated appendicitis.

**Conclusion:**

CRP was observed in our study to be an independent marker of severity in acute appendicitis.

## Highlights

• Evaluation of inflammatory markers is considered helpful in the diagnosis of acute appendicitis.
• C-Reactive Protein (CRP) is considered one of the most important inflammatory markers.
• CRP was helpful not only as an independent marker of severity in acute appendicitis but also predicting complications and perforation as well with higher sensitivity.
• CRP has the highest specificity for complicated appendicitis and the highest positive likelihood ratio for perforated appendicitis.

## 1 Introduction

In pediatrics, acute appendicitis is the most common surgical emergency [1]. Complications related to appendicitis are a major clinical burden in surgical care [2]. It is important to promptly assess the severity of appendicitis and we have different hematological and radiological parameters that can give an early estimate of disease burden. However, clinical assessment with a thorough history and physical examination remains vital to evaluate the patients with unconfirmed acute appendicitis [3]. Appendectomy, whether laparoscopic or open is the most frequently performed surgical intervention worldwide [1,3]. Post-surgery, tissue samples are taken to evaluate for histopathology, however many times histology turned out to be negative either due to improper evaluation or resolve inflammatory response. Some studies report a negative appendicectomy rate of 8.2% while other reports as high as 10–15% [3,4].

Evaluation of inflammatory markers is considered helpful in the diagnosis of acute appendicitis [5], however, they should not be only relied upon (especially in HIV-infected patients) [6]. C-Reactive Protein (CRP) is considered one of the most important inflammatory markers [7]. Since it is important to evaluate disease severity for immediate management (conservative or surgical), late diagnosis can proceed towards perforation, peritonitis and sepsis. On the other hand, a misdiagnosed appendectomy can contribute towards avoidable surgical complications like wound infection and adhesions.

CRP was first discovered by Tillet and Francis, produced in the liver in response to acute inflammation and considered an acute phase reactant [8]. It is a non-specific but more sensitive and authentic indicator of inflammatory response than total leukocyte count (TLC) and erythrocyte sedimentation rate (ESR). In acute appendicitis, it increases with the severity of infection [9]. The main objective of this study is to decipher the incline of CRP, TLC and neutrophils in acute appendicitis and their ability to predict complications and perforation in the pediatric age group.

## 2 Materials and methodology

The study was conducted in the Pediatric Surgery Department of a tertiary care hospital, Karachi. It is a 700 bedded hospital with 32 specialty services located in Southern Pakistan. It was designed as a cohort study after approval from the ethical review board. The research is registered with hospital registry of Liaquat National Hospital and Medical College, with a unique identifying number: R.C-LNH-ER-07/2021/74. STROCSS guidelines were adhered while reporting the findings [10]. It included all the patients diagnosed and operated on for acute appendicitis within the pediatric age group for a period of 1 year (from January 1, 2020 to December 31, 2020). The non-probability consecutive sampling method was used to recruit the patients. Pediatric patients of both genders admitted to either pediatric intensive care unit (ICU) or ward having a confirmed clinical and radiological diagnosis of acute appendicitis were included in the final analysis between age 1–15 years. Serum samples for CRP and complete blood pictures were sent for the enrolled patients at admission before moving to the operating table and those with missing laboratory parameters were excluded from the study. CRP was analyzed via Cobas (C-311) using immunoturbidimetric assay (Roche/Hitachi Cobas C systems). The patients who had conservative management were also excluded as we only included patients undergoing appendicectomies. Hence, a total of 135 patients met the inclusion criteria for further analysis.

Data regarding the patient's age, gender, duration of onset of symptoms, pulse rate and temperature were collected from the individual medical records. Data were analyzed via SPSS version 25.0 (IBM Corp., Armonk, NY, USA). Mean, standard deviation and median, interquartile range were reported for descriptive variables. P-values were calculated by student's t-test and Mann Whitney *U* test accordingly. The rest were presented as frequency and percentage. Diagnostic accuracy of laboratory parameters was quantified using the Receiver operating characteristic (ROC) analysis. The optimum cut off values were determined separately for complicated appendicitis group (including simple perforated appendicitis, gangrenous with perforation, suppurative with perforation and catarrhal appendicitis) and subsequently for perforated group (including simple perforated appendicitis, gangrenous with perforation and suppurative with perforation), using area under the curve (AUC) and formulated a 2 × 2 contingency table to calculate appropriate sensitivity, specificity, positive predictive value, negative predictive value, positive likelihood ratio, negative likelihood ratio and diagnostic accuracy. A p-value of less than 0.05 was considered statistically significant (two-tailed).

## 3 Results

The median (IQR) age of study population was 9 (7–11) years, the majority of which fell into 6–12 years of age group. About 70% were males, and the rest 30% were females. Mean TLC was 15.32 × 10^9^/μL, mean neutrophils count was 79.44% and mean CRP levels were 9.13 mg/dL. Relevant clinical information including pulse rate, onset of symptoms and febrile history is mentioned in Table 1.

**Table 1:** General and clinical characteristics of the study population (n = 135).

Age (years)	Mean (SD): 8.90 (2.814)	Median (IQR): 9.00 (7.00–11.00)
Age range	<6 years: 20 (14.8%)	
	6–12 years: 100 (74.1%)	
	>12 years: 15 (11.1%)	
Onset of symptoms (hours)	Mean (SD): 43.14 (33.620)	Median (IQR): 24.00 (24.00–48.00)
Pulse rate (per minute)	Mean (SD): 107.90 (13.910)	Median (IQR): 110.00 (100.00–120.00)
TLC (x10^9^/μL)	Mean (SD): 15.32 (6.202)	Median (IQR): 14.30 (11.50–19.50)
Neutrophils (%)	Mean (SD): 79.44 (9.083)	Median (IQR): 80.00 (79.00–86.00)
CRP (mg/dL)	Mean (SD): 9.13 (18.046)	Median (IQR): 2.94 (0.63–13.00)
Gender	Male: 94 (69.6%)	Female: 41 (30.4%)
Fever	Febrile: 29 (21.5%)	Afebrile: 106 (78.5%)
Histopathology	Positive: 127 (94.1%)	Negative: 8 (5.9%)
Histopathological classification of appendicitis	Simple Appendicitis: 62 (45.9%)	Complicated appendicitis: 65 (48.1%)
Histopathological classification based on perforation	Non-perforated: 90 (66.7%)	Perforated: 37 (27.4%)
Post-operative complications	None: 121 (89.6%)	
	Wound infection: 9 (6.7%)	
	Intra-abdominal collection: 4 (3.0%)	
	Adhesive small bowel obstruction: 1 (0.7%)	

CRP: C-reactive protein; TLC: total leukocyte count; SD: standard deviation; IQR: interquartile range.

Histopathology came out positive in 127 individuals and the rest 5.9% (n = 8) patients had negative histopathology. Out of those 127 patients, 62 had simple appendicitis and the rest 65 had complicated appendicitis. Out of those 65 complicated patients, 37 had shown perforated appendicitis with only a few of them shown post-operative complications like wound infection (6.7%), intra-abdominal collection (3.0%) and bowel obstruction (0.7%). The majority of individuals had suppurative appendicitis on histopathology (35.6%) followed by catarrhal (20.7%), simple perforated (18.5%) and interstitial appendicitis (7.4%). Gangrene was observed in only 4.4% of perforated appendices as shown in Table 2.

**Table 2:** Varying histopathological presentation of acute appendicitis on biopsy (n = 135).

a Normal histopathology	8 (5.9%)
a Simple appendicitis (phlegmonous)	4 (3.0%)
b Catarrhal appendicitis	28 (20.7%)
a Interstitial appendicitis	10 (7.4%)
a Suppurative appendicitis	48 (35.6%)
b Simple perforated appendicitis	25 (18.5%)
b Gangrenous with perforation	6 (4.4%)
b Suppurative with perforation	6 (4.4%)

a Normal histopathology, Simple appendicitis, Interstitial appendicitis and Suppurative appendicitis were grouped together as Simple appendicitis.

b Simple perforated appendicitis, Gangrenous with perforation, Suppurative with perforation and Catarrhal appendicitis were interpretated as Complicated Appendicitis.

**Fig. 1:**
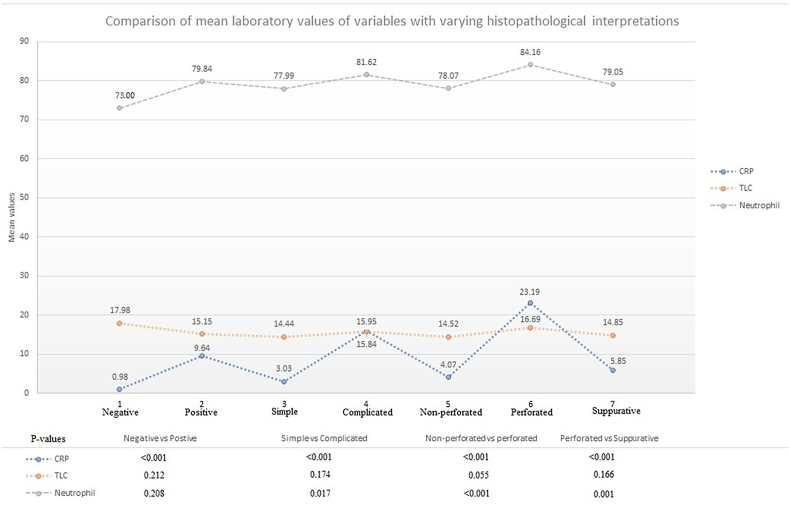
Comparative analysis between varying histopathological presentations of acute appendicitis and laboratory markers (Student's t-test).

**Fig. 2:**
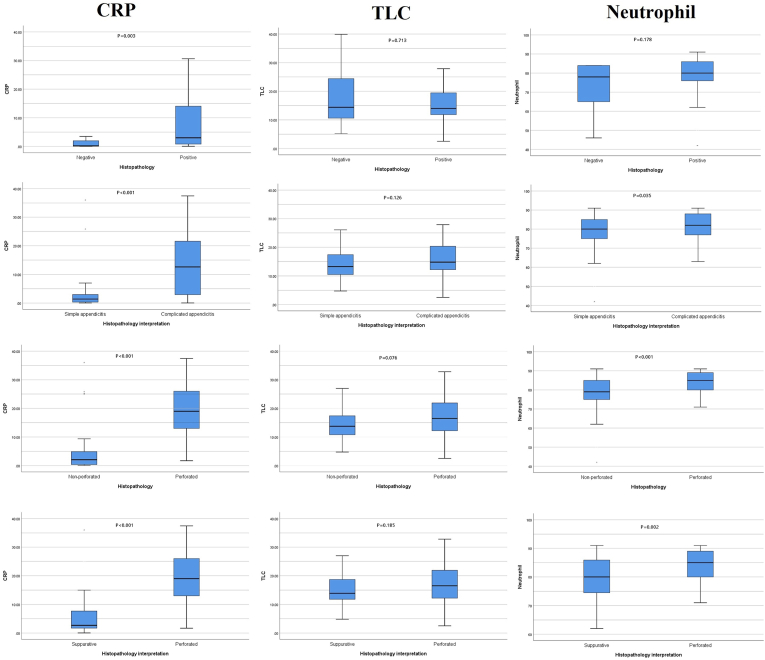
Box plots of a non-parametric distribution of laboratory markers showing medians and respective interquartile ranges (Mann Whitney *U* test).

When comparing these markers with individual histopathological presentations, CRP was found more predictive of complicated (P < 0.001) and perforated appendicitis (P < 0.001) when compared with simple, non-perforated and suppurative appendicitis. Negative histopathology was also successfully predicted by CRP (P = 0.003) however, neutrophil counts and TLC were not found discriminative in this regard as shown in Fig. 1. Neutrophil count further discriminated between simple and complicated appendicitis (P = 0.017), non-perforated vs perforated appendicitis (P < 0.001), and perforated vs suppurative appendicitis (P = 0.001). TLC was not found significant in any category. Fig. 2 represents box plots with a non-parametric distribution of studied markers among individual histopathological presentations.

**Fig. 3:**
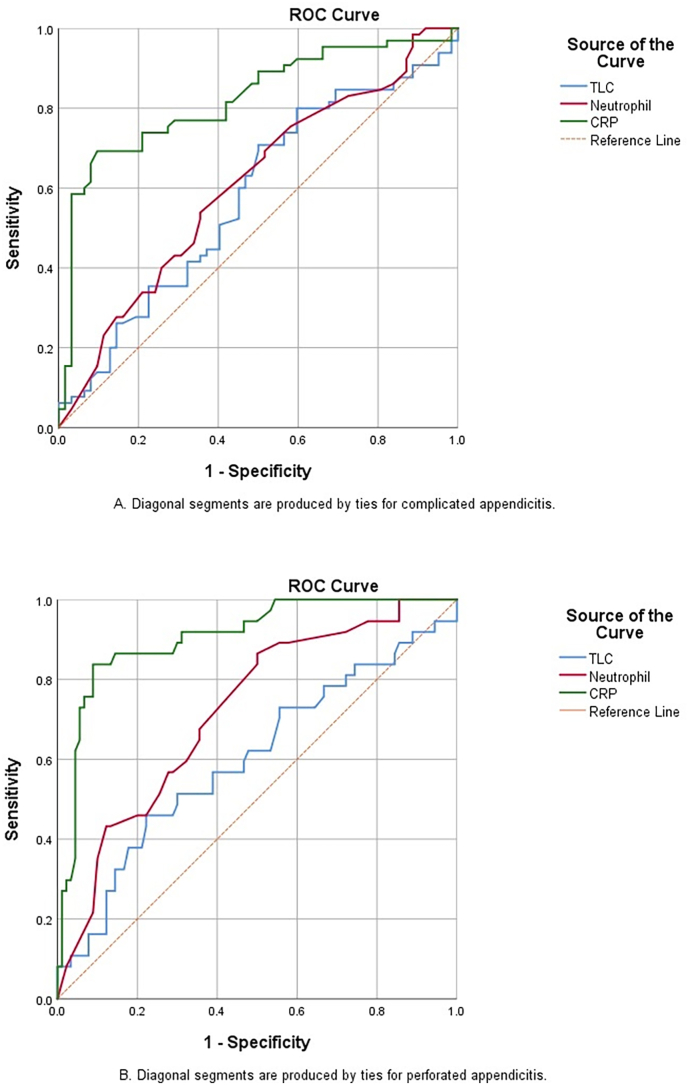
Receiver operating characteristic curves for CRP, TLC and neutrophils in complicated appendicitis (A) and perforated appendicitis (B).

Receiver operating analysis was conducted separately on complicated appendicitis patients (n = 65) followed by exclusively perforated appendicitis patients (n = 37). CRP was able to demonstrate perforation with higher sensitivity and specificity than complicated appendicitis only. Furthermore, CRP was a better marker than neutrophils and TLC in predicting both perforated and complicated appendicitis. TLC was not found statistically significant in predicting both complicated and perforated appendices. The cut off value of TLC was 13.20 × 10^9^/μL, at which it was 70% sensitive for complicated appendicitis and 73% sensitive for perforation, however, accuracy was quite low (60% for complicated and 52% for perforated appendicitis) as shown in Table 3. Neutrophil counts had a higher sensitivity and negative predictive value for perforated appendicitis but higher specificity for complicated appendicitis. Among the three markers, CRP has the highest specificity for complicated appendicitis and the highest positive likelihood ratio for both complicated and perforated appendicitis with good accuracy (79.5% and 88.9% respectively). The corresponding AUC were 0.821 and 0.907 respectively as shown in Table 4 and Fig. 3.

**Table 3:** Receiver operating characteristics analysis of laboratory markers for complicated appendicitis.

Variables	TLC (× 10^9^/μL)	Neutrophils (%)	CRP (mg/dL)
Cut-off value	13.20	80.50	6.70
AUC	0.579	0.608	0.821
95% confidence interval	0.479–0.679	0.510–0.706	0.745–0.896
Sensitivity (%)	70.8	53.8	69.2
Specificity (%)	50.0	64.5	90.3
Positive predictive value (%)	59.7	61.4	88.2
Negative predictive value (%)	62.0	57.1	73.7
Positive likelihood ratio	1.416	0.834	7.134
Negative likelihood ratio	0.584	0.716	0.341
Accuracy (%)	60.6	59.1	79.5
Youden index	1.193	0.173	0.585
Standard error	0.051	0.050	0.038
P-value	0.126	0.035	<0.001

AUC: area under the curve; TLC: total leukocyte count; CRP: C-reactive protein.

**Table 4:** Receiver operating characteristics analysis of laboratory markers for perforated appendicitis.

Variables	TLC (x10^9^/μL)	Neutrophils (%)	CRP (mg/dL)
Cut-off value	13.20	79.50	9.69
AUC	0.600	0.721	0.907
95% confidence interval	0.487–0.714	0.626–0.815	0.851–0.964
Sensitivity (%)	73.0	83.8	83.8
Specificity (%)	44.4	50.0	91.1
Positive predictive value (%)	35.1	40.8	79.5
Negative predictive value (%)	80.0	88.2	93.2
Positive likelihood ratio	1.312	1.676	9.415
Negative likelihood ratio	0.608	0.324	0.177
Youden index	1.164	1.328	0.739
Accuracy (%)	52.7	59.8	88.9
Standard error	0.058	0.048	0.029
P-value	0.076	<0.001	<0.001

AUC: area under the curve; TLC: total leukocyte count; CRP: C-reactive protein.

## 4 Discussion

Previously, studies have been conducted to affirm the association of CRP with complicated appendicitis. Lai et al. included 44 adult patients in their analysis and found out 31 patients had complicated appendicitis, in whom CRP >4.01 mg/dL was predictive with 71% sensitivity and 100% specificity [11]. In our study, CRP cut off was higher, however, still able to predict complicated appendicitis with 79.5% accuracy, and a similar positive predictive value and positive likelihood ratio. Further, TLC was not found associated with complicated appendicitis, a finding similar to our study [11]. Another study from India conducted on 65 patients found TLC not helpful for differentiating between appendicitis and negative appendicitis (AUC: 0.679) while associating CRP with AUC of 0.716 at cut off 8.76 mg/dL [12]. They, however, had shown lower sensitivity and specificity than our results. Kaya et al. considered both TLC and CRP to have diagnostic value but TLC had lower specificity [13]. Moreover, CRP levels were able to differentiate between phlegmonous appendicitis and perforated appendicitis at a cut off 1.3 mg/dL with 55.3% sensitivity and 100% specificity, which is similar to our findings at cut off 9.69 mg/dL [13]. A study conducted on pediatric population concluded a higher odd of perforated appendicitis with raised TLC, neutrophils combined with CRP levels [14]. CRP value > 6.15 mg/L has a sensitivity of 100.0% and a specificity of 54% in predicting complicated appendicitis according to another study [15], with a positive predictive value of 100% and a negative predictive value of 61.54%, similar to our results. A study from Taiwan had used different cut off values of CRP on the first 3 days after the onset of symptoms and concluded that serial measurements may serve as a useful predictive parameter in the early diagnosis of perforated appendicitis [16].

The proportion of negative histopathology seen in our study (5.9%) was also observed in another study conducted on 54 patients [17]. CRP >84 mg/L was significantly associated with gangrenous appendicitis in the same study [17]. A study from India was conducted on a similar pattern and concluded TLC having a sensitivity of 97%, specificity of 55% and a positive predictive value of 91% [8]. CRP had a sensitivity of 95%, specificity of 77% and positive predictive value of 95%, while neutrophil count had a sensitivity of 98%, specificity of 38% and positive predictive value of 89% [18]. However, in our analysis of 135 patients, we did not find an association of TLC, but neutrophils and CRP were associated with both complicated and perforated appendicitis. Another study in the pediatric age group contradicted our findings of CRP being a superior marker of complicated and perforated appendicitis [19]. They concluded TLC being more useful in predicting acute appendicitis at histology than CRP, with TLC having a sensitivity of 25%, specificity of 94% and positive predictive value of 88%, while CRP yielded a sensitivity of 52%, specificity of 91 and positive predictive value of 91% [19]. Many studies supported combined evaluations of leucocytes and CRP for being helpful in diagnosis because this increases their positive predictive value when measured together [14,18,20]. But Hodgkinson et al. opposed this finding by claiming that combining both markers has no impact on positive likelihood [19]. Another study concluded that increased CRP is not a definite indicator of acute appendicitis. However, if CRP levels 12 h after the onset of symptoms are <2.5 mg/dL, acute appendicitis can be excluded [21]. A study conducted in Japan on 150 patients has shown that CRP was independently associated with gangrenous appendicitis with a sensitivity of 84.3%, specificity of 75.8%, positive predictive value of 64.2% and negative predictive value of 90.4% at cut off value of 4.95 mg/dL [22]. In our results, complicated appendicitis was predicted by CRP at a much higher specificity of 90.3%. Raja et al. studied 100 patients and found high prevalence of suppurative appendicitis (34%) which is similar to our findings (35.7%) [23]. They had perforations in 13% of cases while we report 27.4% of perforated appendicitis in our cohort [23].

A study from Egypt analyzed 100 patients of appendicitis among whom 25 had a complicated disease. TLC has a sensitivity of 85%, specificity of 75%, positive predictive value of 44% and negative predictive value of 100% in predicting acute appendicitis while CRP has a sensitivity of 93.3%, specificity of 86.6 positive predictive value of 55% and negative predictive value of 88% [24]. Lastly, a study conducted on 197 patients in Turkey, concluded no association of raised CRP levels with appendicitis as opposed to neutrophil counts and TLC which were found associated [25]. TLC at a cut off value > 11 was predictive with AUC: 0.73 at a sensitivity of 73%, specificity of 60%, positive predictive value of 75%, negative predictive value of 57% and accuracy of 73%. We had also predicted similar values for TLC at cut off >13.20 but with a lower AUC and accuracy reported [25]. Neutrophil count was predictive with an AUC of 0.75 at a sensitivity of 72%, specificity of 67%, positive predictive value of 72%, negative predictive value of 60% and accuracy of 70% [25]. While we reported an AUC of 0.608 for neutrophils with an accuracy of 59%.

There were few limitations of our study, including a single-center analysis and limited sample size. There was no randomization of study groups, for instance, no control group was considered to have a normal appendix. The analysis was between complicated and non-complicated appendicitis which was the strength of the study.

## 5 Conclusion

CRP was well observed in our study to be an independent marker of severity in acute appendicitis with not only predicting complications but perforation as well with higher sensitivity. TLC was not significantly associated with severe appendicitis as opposed to neutrophil count which was found significantly associated with both perforated and complicated appendicitis. Further large-scale studies should be carried to find out the longitudinal association of these markers with the clinical outcomes as our study was not able to follow up the patients with serial evaluation of these markers.

## Provenance and peer review

Not commissioned, externally peer reviewed.

## Funding sources

No funding required for the study.

## Ethical approval statement

Ethical approval was taken in this study from institutional review board (Ref:App.#R.C-LNH-ER-07/2021/74), and consent to participate was not required due to retrospective nature of the study.

## Data availability statement

All data will be made available on a reasonable request to the corresponding author.

## Consent

Consent to participate was not required due to retrospective nature of the study.

## Author contribution

S.J.H·K, M.S.A, and S.T conceived the idea, A, M.A.S, F·Y, and M.J.T retrieved the data, did write up of letter, and finally, T.A.K, M.A, and Y.A reviewed and provided inputs. All authors approved the final version of the manuscript.

## Registration of research studies

1. Name of the registry: Liaquat National Hospital and Medical College.
2. Unique Identifying number or registration ID: R.C-LNH-ER-07/2021/74
3. Hyperlink to your specific registration (must be publicly accessible and will be checked):

## Guarantor

Muhammad Sohaib Asghar and Muhammad Junaid Tahir.

## Declaration of competing interest

The authors have no conflicts of interest to declare.
